# STAT3 Relays a Differential Response to Melanoma-Associated *NRAS* Mutations

**DOI:** 10.3390/cancers12010119

**Published:** 2020-01-02

**Authors:** James Kim, Daniel Novak, Christos Sachpekidis, Jochen Utikal, Lionel Larribère

**Affiliations:** 1 Skin Cancer Unit, German Cancer Research Center (DKFZ), 69120 Heidelberg, Germany; j.kim@dkfz-heidelberg.de (J.K.); d.novak@dkfz-Heidelberg.de (D.N.); j.utikal@dkfz-Heidelberg.de (J.U.); 2 Department of Dermatology, Venereology and Allergology, University Medical Center Mannheim, Ruprecht-Karl University of Heidelberg, 68167 Mannheim, Germany; 3 Clinical Cooperation Unit Nuclear Medicine, German Cancer Research Center (DKFZ), 69120 Heidelberg, Germany; c.sachpekidis@dkfz-heidelberg.de

**Keywords:** NRAS, mutation, melanoma, oncogene-induced senescence, STAT3

## Abstract

Melanoma patients carrying an oncogenic *NRAS* mutation represent 20% of all cases and present worse survival, relapse rate and therapy response than patients with wild type *NRAS* or with *BRAF* mutations. Nevertheless, no efficient targeted therapy has emerged so far for this group of patients in comparison with the classical combination of BRAF and MEK inhibitors for the patient group carrying a *BRAF* mutation. NRAS key downstream actors should therefore be identified for drug targeting, possibly in combination with MEK inhibitors. Here, we investigated the influence of different melanoma-associated *NRAS* mutations (codon 12, 13 or 61) on several parameters such as oncogene-induced senescence, cell proliferation, migration or colony formation in immortalized melanocytes and in melanoma cell lines. We identified AXL/STAT3 axis as a main regulator of *NRASQ61*–induced oncogene-induced senescence (OIS) and observed that *NRASQ61* mutations are not only more tumorigenic than *NRASG12/13* mutations but also associated to STAT3 activation. In conclusion, these data bring new evidence of the potential tumorigenic role of STAT3 in *NRAS*-mutant melanomas and will help improving current therapy strategies for this particular patient group.

## 1. Introduction

Malignant melanoma, which originates from melanocytes, has the highest mortality rate (48%) among skin cancers because of its very early and aggressive formation of metastasis [1,2,3]. Its mutational status harbors the most genetic mutations compared to other cancer types, which has an impact both on understanding tumor biology and treatment options [4]. The most common mutation is found in the oncogene *BRAF* (50%) followed by mutations in the *NRAS* gene (20%) [5,6]. *NRAS*-mutant melanomas form thicker tumors and have a higher mitotic rate than *NRAS*-wildtype melanomas [7,8,9]. Interestingly, *NRAS*-mutant melanoma patients show worse survival, relapse rate and therapy response than patients with wild type *NRAS* or with *BRAF* mutations [9].

Whereas *BRAF* mutated melanomas have efficient targeted treatment options with *BRAF*-inhibitors (vemurafenib, dabrafenib and encorafenib) in combination with MEK-inhibitors (cobimetinib, trametinib and binimetinib), *NRAS* was thought to be an “undruggable” target due to missing FDA-approved targeted therapies available [10,11,12,13,14,15,16]. As targeting *NRAS* directly is not yet possible, there are different promising approaches with MEK inhibitors combined with other drugs targeting downstream and upstream signalings. A phase III trial (NEMO) comparing binimetinib to dacarbazine therapy on *NRAS*-mutated patients showed promising results with an increased median progression-free survival of 2.8 months (95% CI 2.8–3.6) in the binimetinib group compared to 1.5 months (1.5–1.7) in the dacarbazine group (hazard ratio 0.62 (95% CI 0.47–0.80); one-sided *p* < 0.001) but not in overall survival [17]. Recently, a preclinical study has described a new combination strategy involving BET inhibitors with MEK inhibitors to overcome drug resistance in NRAS-mutant melanoma [18]. More recently, new oncogene-targeting chemotherapeutic agents have shown promising effects especially in tumors mutated on *KRAS*, *NRAS* and *BRAF* including melanoma [19].

Mechanistically, most *NRAS* mutations lead to a constitutively active form of this GTPase, altering downstream signaling pathways and influencing cellular proliferation, differentiation and survival [20]. At the *NRAS* locus site, mutations are found in codon 61 almost exclusively rather than in codon 12 or 13 although they all possess an oncogenic activity [21]. The reason why such a discrepancy in mutations’ frequency exists is not yet fully understood, but codon mutational status throughout diverse cancer entities has clearly important clinical implications, e.g., different therapy responses to cetuximab therapy in colorectal cancer or prognostic relevance in non-small-cell lung cancer [22,23,24]. For instance, *NRASQ61* mutations were described to induce greater melanoma formation than *NRASG12* mutations in murine cells but the underlying mechanism is not quite clear [25].

Mutations in oncogenes such as *RAS* are known to induce a prolonged and irreversible arrest in primary mammalian cells, so called oncogene-induced senescence (OIS) as a mechanism of tumor suppression [26,27,28,29]. The induction of OIS is usually marked by senescence-associated heterochromatin foci (SAHF), which are alterations in the chromatin structure, repressing the expression of genes involved in proliferation as a result of distinct histone modifications [30]. OIS can also be visualized by the senescence-associated-β-galactosidase activity (SA-β-Gal). Therefore further cooperating genetic alterations are needed to override OIS and induce tumor formation [31]. Indeed, a cooperation between *NF1* mutations and *BRAF* mutations was described to overcome OIS and to affect the melanoma response to targeted therapies [32,33].

More recently, *NRAS* secondary mutations were described to be responsible for the development of drug resistance in *BRAF*-mutated patients [12,34].

Therefore, it is of crucial importance to determine what signaling pathways in addition to MAPK are specifically involved in *NRAS*-mutated tumors. In this study, we focused on comparing the impact of *NRAS* mutations on codon 61 with these on codon 12/13 in the melanocytic lineage. We found that *NRASG12/13* mutants induce a stronger OIS-associated phenotype than *NRASQ61* mutants in melanocytes. We also identified AXL/STAT3 axis as a key regulator of *NRASQ61*–induced OIS. Moreover, we showed that *NRASQ61* mutations have greater tumorigenic potential than *NRASG12/13* both in immortalized melanocytes and in human melanoma cell lines through activation of the STAT3 pathway.

## 2. Results

### 2.1. NRAS^G12/13^ Mutants Induce a Stronger OIS-Associated Phenotype than NRAS^Q61^ Mutants in Normal Human Melanocytes (NHM)

**Figure 1:**
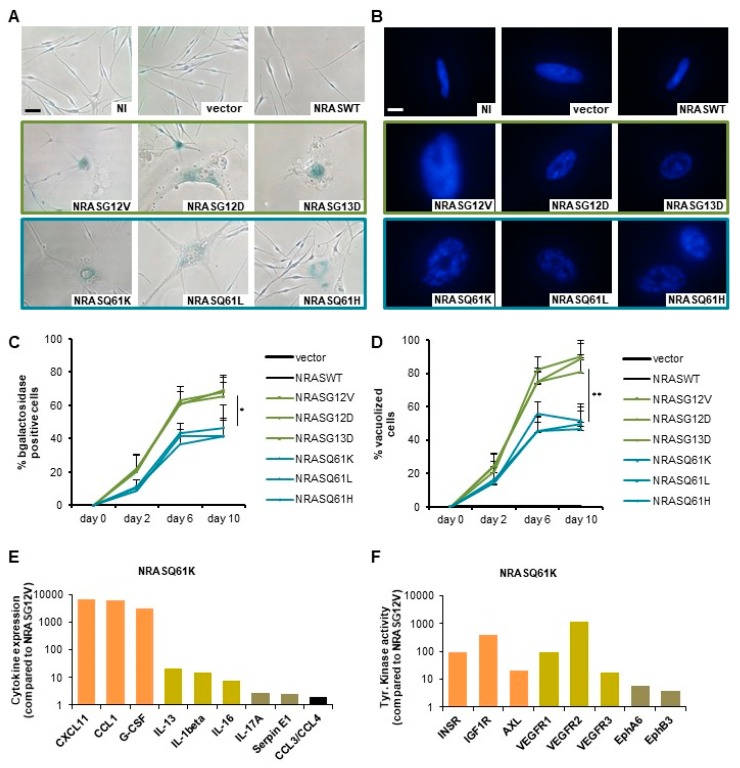
*NRAS^G12/13^* mutants induce a stronger oncogene-induced senescence (OIS)-associated phenotype than *NRAS^Q61^* mutants in normal human melanocytes (NHMs). (**A**) Normal human melanocytes (NHM) expressing indicated *NRAS* mutants were subjected to SA-β-Gal staining after 9 days. NI: non infected, vector: empty vector. Scale bar: 100 µm. (**B**) DAPI nuclear staining of NHM expressing indicated *NRAS* mutants shows an accumulation of OIS-associated heterochromatin foci (SAHF) with enlarged punctuated nuclei. Scale bar: 20 μm. (**C**) Quantification of senescence-associated SA-β-Gal positive cells in percent. (**D**) Quantification of vacuolized cells in percent. (**E**) Protein expression of OIS-associated cytokines as a fold change to *NRASG12V*. (**F**) Tyrosine kinase activity as a fold change to *NRASG12V*. *p* values from three independent experiments by two-tailed, unpaired sample *t* test (* *p* < 0.05, ** *p* < 0.01, *** *p* < 0.005).

**Figure 2:**
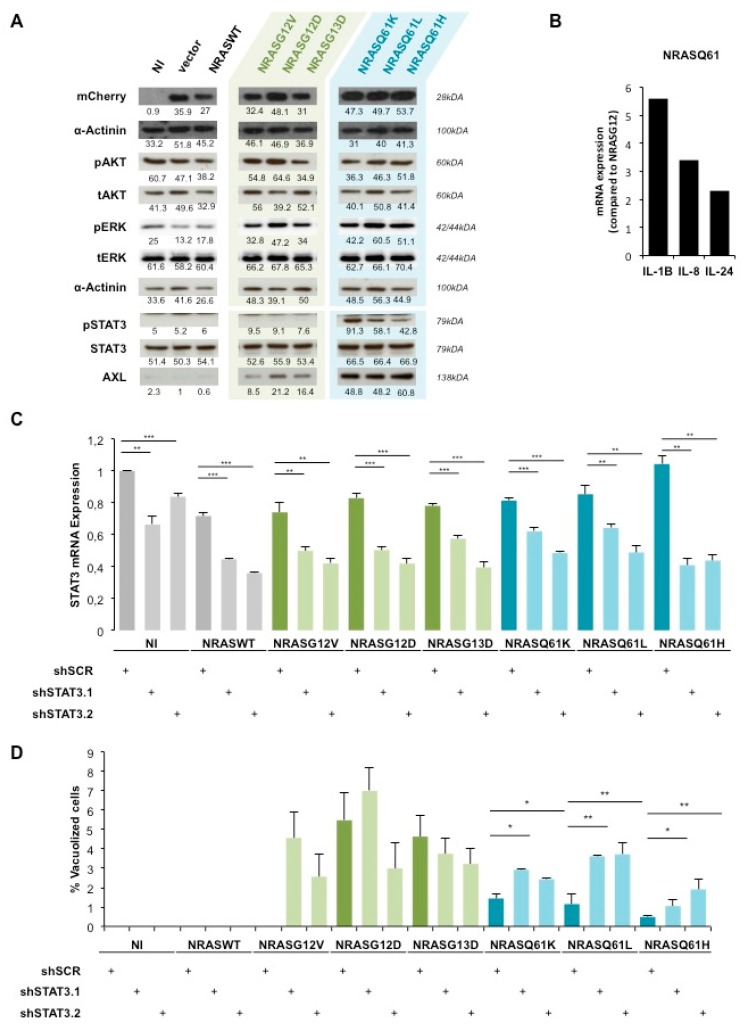
AXL/STAT3 axis is a key regulator of *NRASQ61*—induced OIS in NHM. (**A**) Western Blot analysis of AKT, ERK and STAT3 activation status in NHM expressing indicated *NRAS* mutants. NI: non infected, vector: empty vector. (**B**) qPCR analysis of IL-24, IL-1B and IL-8 mRNA levels in NHM expressing indicated *NRAS* mutants. Values were normalized to *NRASG12/13* and shown as fold change. (**C**) mRNA analysis of STAT3 in NHM after double transduction with shSTAT3 (shSTAT3.1, shSTAT3.2) and *NRAS* mutants. (**D**) Quantification of vacuolized cells in percent in the same conditions as in (**C**). * *p* < 0.05, ** *p* < 0.01. *** *p* < 0.001.

We first investigated the effect of *NRAS* mutations on the induction of OIS in normal human melanocytes (NHMs). The expression of mutated *NRAS* led to different intensity of OIS when compared to control conditions with an empty vector or with *NRASWT,* as shown by flattened cell morphology and accumulation of OIS-associated heterochromatin foci (SAHF; Figure 1A,B). Indeed, the quantification of senescence-associated-β-galactosidase activity (SA-β-Gal) showed up to 69% positive cells by day 10 after transduction with *NRASG12V, NRASG12D* and *NRASG13D* but only up to 46% positive cells after transduction with *NRASQ61K, NRASQ61L* and *NRASQ61H* (Figure 1A,C). Similarly, the quantification of vacuolized cells showed up to 90% in the group of mutations *NRASG12/13* but only up to 51% in the group of mutations *NRASQ61* (Figure 1D). To investigate the mechanisms lying behind the observed OIS, we analyzed the secretome in the cells’ supernatants, as well as the activity of a set of kinases. Indeed, previous studies described the senescence-associated secretome as a set of cytokines, which can regulate the senescence via an auto and paracrine loop [35,36]. In addition, several kinases including AXL were described to be involved in the OIS [37]. In our experiments, we observed a significant upregulation of a cytokine panel (including IL-8, IL-24 and IL-1β) in a gene expression profiling of these cells, which was verified by elevated protein secretion in the supernatants (Figure 1E and Figure 2B). On top of that, increased tyrosine kinase activity (including INSR, IGFR1, VEGFR and AXL) in *NRASQ61* cells compared to *NRASG12/13* cells was visible (Figure 1F).

Therefore, these data show that *NRASG12/13* mutants induce a stronger OIS-associated phenotype than *NRASQ61* mutants in NHM, which is associated with specific cytokines expression and kinases activation.

### 2.2. AXL/STAT3 Axis Is a Key Regulator of NRAS^Q61^—Induced OIS in NHM

To unravel the underlying signaling pathways involved in the occurrence of mutant *NRAS*-induced OIS, we first analyzed the activation status of MAPK and PI3K signaling by western blot (Figure 2A).

ERK was weakly phosphorylated in the non-infected (NI), empty vector (vector) and *NRASWT* conditions. Interestingly, this phosphorylation increased in a similar manner among all *NRAS* mutants whereas the total ERK expression was unchanged in all conditions. AKT phosphorylation as well as total AKT expression was also unchanged in all conditions. α-Actinin was used as a loading control and mCherry expression allowed us to verify equal *NRAS* transgenes expression.

Therefore, to identify other involved pathways we performed a global gene expression analysis between *NRASG12/13* and *NRASQ61* NHM. Among the top regulated genes, we identified several STAT3-inducing interleukins such as IL-8, IL-24 and IL-1β [38,39]. We confirmed a two-fold upregulation of IL-24, a five-fold upregulation of IL-1β and a three-fold upregulation of IL-8 in *NRASQ61* cells compared to *NRASG12/13* cells by qPCR (Figure 2B). In addition, the western blot analysis showed high phosphorylation of STAT3, specifically in *NRASQ61* cells when compared to *NRASG12/13* cells. As a control, we observed no changes in the total expression of STAT3 (Figure 2A). Moreover, the expression of STAT3-inducer tyrosine kinase AXL was highly increased in *NRASQ61* cells when compared to *NRASG12/13*. These findings strongly suggest an activation of STAT3 signaling, specifically by *NRASQ61* mutations.

To confirm the involvement of STAT3 in mutant *NRAS*-induced OIS, we performed loss-of-function experiments. For this purpose, we double transduced NHM with *NRAS* mutants and with two shRNAs against *STAT3* (shSTAT3.1 and shSTAT3.2). The silencing led to a significant decrease of STAT3 mRNA expression in all conditions (Figure 2C). Indeed, STAT3 silencing efficiency reached an average of 40% mRNA reduction with both shRNAs.

Further, the quantification of vacuolized cells indicated that *STAT3* silencing in *NRASG12/13* cells induced non reproducible and therefore non-significant variations compared to the respective non-targeting shRNA control sample (shSCR). However, we observed a significant 2 to 3-fold change increase of vacuolization in *NRASQ61* cells under *STAT3* silencing (Figure 2D).

Taken together, our data demonstrate a specific activation of AXL-STAT3 signaling by *NRASQ61* leading to lower occurrence of OIS compared to *NRASG12/13* in NHM.

### 2.3. STAT3 Is Involved in NRAS-Driven Migration and Colony Formation of Immortalized Melanocytes (MelSTV)

**Figure 3:**
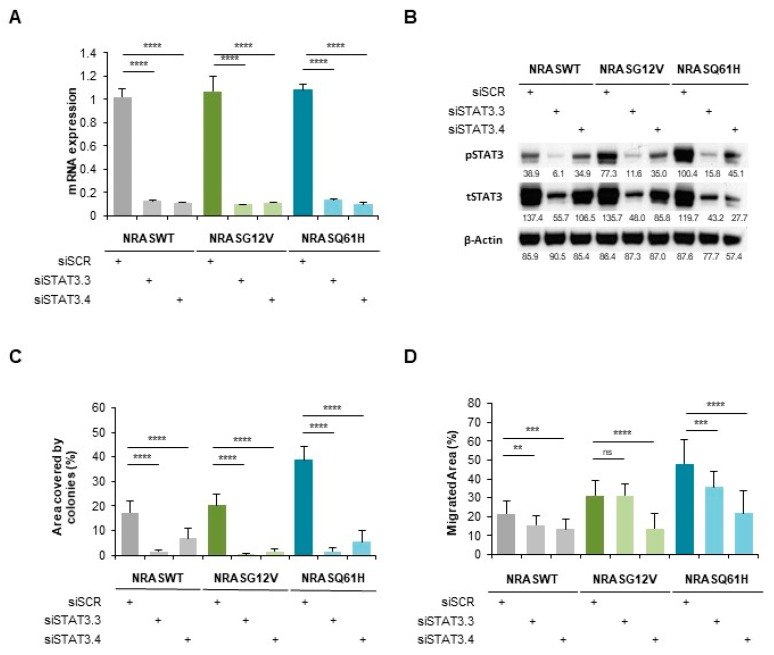
STAT3 is involved in *NRAS*-driven migration and colony formation of immortalized melanocytes MelSTV. (**A**) mRNA expression of *STAT3* in immortalized melanocytes MelSTV expressing either *NRASWT*, *NRASG12V* or *NRASQ61H*, after 48 h transfection with siSTAT3 (siSTAT3.3 and siSTAT3.4). (**B**) Phospho-STAT3 and STAT3 levels shown by Western Blot in the same conditions as in A. (**C**) Colony formation assay of MelSTV in the same conditions as in A. (**D**) Migration of MelSTV in the same conditions as in A. ** *p* < 0.01, *** *p* < 0.001, **** *p* < 0.0001.

Next, we used immortalized melanocytes MelSTV to further study the role of STAT3 in the transforming effects of *NRAS* mutants. After stable transduction with *NRASWT*, *NRASG12V* or *NRASQ61H,* the cells were enriched for the expression of reporter gene mCherry by FACS. The first functional assays showed a significantly enhanced proliferation rate, enhanced migration rate and enhanced colony formation ability of *NRASQ61H* MelSTV compared to *NRASWT* and *NRASG12V* MelSTV (Figure S1). These cells were then transfected for 48 h with siSTAT3 (siSTAT3.1 and siSTAT3.2) or a control siRNA (siSCR). The silencing efficiency at the mRNA level reached around 90% with both siRNAs in all three *NRAS* conditions (Figure 3A). Accordingly, *STAT3* silencing with either siRNA also led to a strong reduction of STAT3 protein expression (62% and 42% respectively; Figure 3B). Of note, in this cell line like in NHM, *NRASQ61H* strongly increased STAT3 phosphorylation level compared to *NRASG12V* or to *NRASWT*. However, *STAT3* silencing significantly reduced STAT3 phosphorylation to a similar basal level in all three *NRAS* conditions.

A colony formation assay indicated that *NRASQ61H*-induced colony increase (two-times more when compared to *NRASG12V*) was mostly abolished by *STAT3* silencing. This effect was also observed with *NRASG12V* and *NRASWT* (Figure 3C).

Moreover, we performed a scratch-like experiment on these cells, which presented a higher migration rate of *NRASQ61H* cells than that of *NRASG12V* and *NRASWT* cells (Figure 3D). However, and in accordance to the colony formation assay, *STAT3* silencing strongly reduced the migration rate in all three *NRAS* conditions. Nevertheless, *STAT3* silencing indicated a similar involvement of STAT3 in both *NRASQ61H* and *NRASG12V* phenotypes.

These data show that STAT3 is a key actor of *NRAS*-driven migration and colony formation of MelSTV, independently of their *NRAS* mutational status.

### 2.4. NRAS^Q61H^ is more Tumorigenic than NRAS^G12V^ and Activates STAT3 

**Figure 4:**
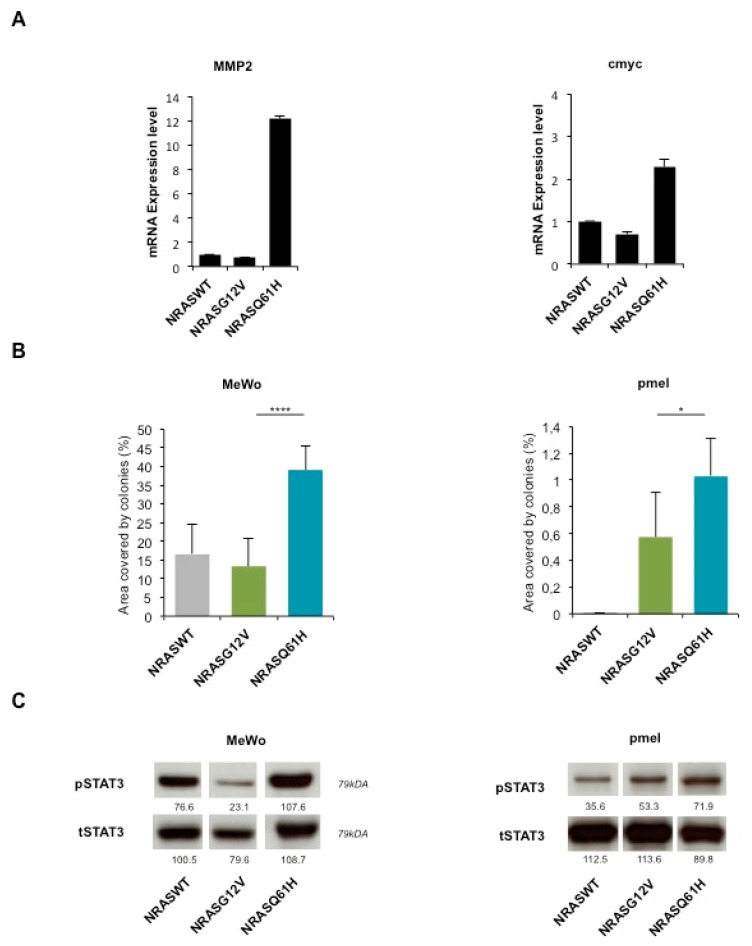
*NRASQ61H* is more tumorigenic than *NRASG12V* and activates *STAT3.* (**A**) mRNA expression levels of STAT3 target genes *MMP2* and *cMYC* in MelSTV. (**B**) Colony formation assay of melanoma cell lines Mewo, and immortalized melanocytes pmel expressing either *NRASWT*, *NRASG12V* or *NRASQ61H*. (**C**) Western blot shows respective expression of P-STAT3 and total STAT3. * *p* < 0.05, **** *p* < 0.0001.

Interestingly, STAT3 targets *MMP2* and c*MYC*, were upregulated in *NRASQ61H* cells compared to NRASG12V or *NRASWT* cells, concordant with STAT3 activation specifically by this mutant (Figure 4A).

Furthermore, we tested the colony formation ability after transduction with *NRASQ61H, NRASG12V* or *NRASWT* (Figure 4B). As in MelSTV, the colony formation of melanoma cell line MeWo and immortalized melanocytes pmel was higher with *NRASQ61H* than with the two other conditions. This effect correlated with an increased STAT3 phosphorylation (as a control, total STAT3 expression was unchanged) as observed by western blot (Figure 4C).

These results indicate that *NRASQ61H* induces a stronger tumorigenic phenotype in immortalized melanocytes and melanoma cell lines when compared with *NRASG12V,* and this effect correlates to an increased STAT3 phosphorylation status.

## 3. Discussion

In this study, we showed that *NRASG12/13* mutants induce a stronger OIS-associated phenotype than *NRASQ61* mutants in primary human melanocytes. This difference may not be caused by variation in protein expression levels (equivalent reporter gene expression) and is unlikely due to RAS activity variations (equal phosphorylation levels of classical RAS downstream targets, ERK and AKT; Figure 2A). Thus, this result strongly suggests a mutant-specific activation of an additional NRAS downstream pathway.

In line with our results, a previous study comparing *NRASQ61R* mutant and *NRASG12D* mutant in a transgenic mouse model of melanoma described little differences in the activation of downstream PI3K or RAF targets. They could establish via biochemical approaches based on nucleotide binding and hydrolysis, a higher activation of *NRASQ61R* protein compared to *NRASG12D* [25]. Additional players such as guanine nucleotide exchange factors (GEF) enhance the GTP-bound active state of NRAS. Conversely, GTPase-activating proteins (GAPs) accelerate the GTP-hydrolysis and favor NRAS GDP-bound inactive state. These regulators of RAS activity may therefore also play a role in the differential phenotype of the *NRAS* mutants. For example, PREX2 GEF activity was activated by mutations found in *NRAS*-mutant melanoma [40]. Moreover, other molecular mechanisms such as posttranslational or epigenetic modifications could also affect the outcome of *NRAS* mutations. Indeed, it was described that mutation-induced epigenetic remodeling cooperates with *NRAS*-mutations to drive myeloid transformation [41].

More recently, another study focused on the proteome of human melanocytes bearing *NRAS* mutations. The authors identified an increased PI3K/AKT activation by *NRASG12V* and an increased MAPK activation with *NRASG61L* [42]. These apparent conflicting data may be explained by different transduction kinetics.

Our data on *NRASQ61*-induced OIS in melanocytes also revealed an association with a specific set of cytokine expression and with specific tyrosine kinase activation (Figure 1E,F). These proinflammatory cytokines are part of the secretome produced by senescent cells. Of note, some of these cytokines are known activators of STAT3 signaling (IL-8, IL-1β and IL-24), which is protumorigenic and favors melanoma reprogramming towards a tumor-initiating phenotype [36]. Thus, this increased production of cytokines could account for the observed lower NRASQ61-induced OIS level when compared to NRASG12 via a paracrine mechanism in vitro. The tyrosine kinase profiling results suggest that a discrete number of kinases is specifically activated by *NRASQ61,* which may lead to protumorigenic signaling and contribute to a reduction of OIS. 

In particular, AXL, IGF1R and VEGFR are described as activators of STAT3 [43,44]. The observed increased activity of these kinases together with STAT3 activation may suggest a molecular link in *NRASQ61* expressing cells (Figure 1F and Figure 2A).

Interestingly, a computer-based prediction model for kinases in a previous study identified an upregulation of CK2α by *NRASQ61*-melanocytes [42]. CK2α being a serine/threonine protein kinase, it could play an additional role in *NRASQ61*-induced senescence. Noticeably, another study linked phosphorylation of STAT3 to activation of CK2α as an upstream event in human glioma cells [45]. The exact role of CK2α in the activation of STAT3 and OIS requires more investigation.

We found that STAT3 silencing leads to a significant increase in *NRASQ61*–induced OIS whereas no significant differences were observed with *NRASG12.* These data confirm the key role of STAT3 specifically in *NRASQ61* melanocytes, which is associated with AXL activation. Further studies are needed to fully understand, which kinase directly phosphorylates STAT3 during *NRASQ61*-induced OIS.

In the context of immortalized melanocytes or human melanoma cell lines, we found that *NRASQ61* had a greater ability to induce proliferation, migration and colony formation than *NRASG12.* This phenotypical difference was associated with a stronger STAT3 activation (and with an upregulation of STAT3 targets MMP2 and cMYC), when compared to *NRASG12* (Figure 4A). In line with our findings cMYC overexpression is able to impair OIS in *NRASQ61R*-mutated melanocytes or by its negative regulator PP2A-B56α [46,47]. These observations confirm the nature of *NRASQ61* mutants to be more oncogenic as well as in vivo data that showed higher nevus and melanoma formation in *NRASQ61R* expressing p16INK4a-deficient mice [25].

However, although both STAT3 expression and phosphorylation were greatly impaired by its gene silencing, the reduced effect on migration and colony formation capacities of MelSTV was not different in *NRASG12V* and *NRASQ61H* mutant conditions. This indicates that although this particular STAT3 phosphorylation (Tyr705) is required for these cells’ tumorigenicity, additional factors could play a role in the *NRASQ61*-specific phenotype. 

In contrast to a high mutation burden of *BRAF* in common acquired nevi, *NRAS* mutations were found most frequently in congenital nevi (80%). When characterizing the *NRAS* mutations in detail, there are exclusively mutations in the codon 61 [48]. The absence of lesions carrying G12/13 *NRAS* mutations may support our findings that *NRASQ61* has a stronger tumorigenic effect and less susceptibility to OIS than *NRASG12/13*. In contrast, another study comparing the mutational status of melanoma and their precursor nevi concludes that there is no significant difference in the frequency of *NRAS* mutations between nevi and melanoma stating that *NRAS* mutational status itself is not a prognostic factor for melanoma formation [49]. However, more investigation is needed to understand the selection of *NRASQ61* mutants in benign primary lesions and their transformative development to malignant melanoma.

As mentioned before, *NRAS* mutations in melanoma tumors are responsible for the development of drug resistance in *BRAF*-mutated patients. Since several reports have shown an effectiveness of STAT3 inhibition cells resistant to BRAF inhibitor, it could be of importance to test the effect of STAT3 inhibitors in *NRAS* mutation-driven drug resistance [50,51,52,53].

## 4. Materials and Methods

### 4.1. Cell Lines 

Human primary melanocytes were isolated from patient’s foreskin biopsies according to the ethical regulation (2010-318N-MA, Ethics committee II, University Medical Center Manheim, Germany). Isolated melanocytes were cultured in 254 Medium (Gibco™, ThermoFisher Scientific, Waltham, MA, USA, M254500) supplemented with Human Melanocyte Growth Supplement (Gibco™, ThermoFisher Scientific, S0025) and 20 μg/mL G418 in the first passages to avoid fibroblast growth. Immortalized Melanocytes MelSTV [54], HEK293 and melanoma cell line MeWo were cultured in DMEM Medium (High Glucose, GlutaMax, Gibco™, ThermoFisher Scientific, 31966047) supplemented with 10% fetal bovine serum (Merck, Darmstadt, Germany, S0115), MEM Non-essential Amino Acid Solution (Sigma-Aldrich^®^, St. Louis, MO, USA, M7145), 1% β-mercaptoethanol, 1% penicillin (100 units/mL) and streptomycin (100 μg/mL). Immortalized melanocytes pmel [55], gift from Prof. Hans R. Widlund, Department of Dermatology, Brigham and Women’s Hospital, Boston, MA, USA, were cultured in Ham’s F10 (Gibco™, ThermoFisher Scientific, 11550043) supplemented with 7% FBS, 1% penicillin-streptomycin (100 units/mL penicillin and 10 mg/mL streptomycin, Sigma-Aldrich^®^, P4333), 0.1 mM IBMX (Sigma-Aldrich^®^, I5879), 50 ng/mL TPA (Sigma-Aldrich^®^, P1585), 1 µM Na3VO4 (Sigma-Aldrich^®^, S6508) and 1 µM dbcAMP (Sigma-Aldrich^®^, D0260). All cells were maintained at 37 °C in a humid incubator with 5% CO_2_.

### 4.2. Lentiviral Transduction 

DNA encoding for *NRAS* wildtype and each mutated in either codon 12/13 (G12V, G12D and G13D) or codon 61 (Q61K, Q61L and Q61H) was cloned into a plasmid under control of an EF1α-Promoter. The *NRAS* gene was coupled with the fluorescent reporter-protein mCherry linked with an internal ribosome entry site (IRES). Lentivirus particles were produced using X-tremeGENE™ 9 DNA Transfection Reagent (Roche Applied Science, Mannheim, Germany, XTG9-RO) in HEK293 cells according to the manufacturer’s instructions. Therefore, the virus containing supernatant was collected three times after 12 h each. Cells were transduced with virus supernatant and 4 µg/mL Polybren two times for each 24 h and then cultured in the corresponding culturing medium. Cells were then sorted for mCherry positive cells by flow cytometry.

### 4.3. siRNA/shRNA

siRNA transfection of cells was performed using Lipofectamine RNAiMAX Transfection Reagent (ThermoFisher Scientific, 13778100) resolved in OptiMEM (Gibco™, ThermoFisher Scientific, 31985062) according to the manufacturer’s protocol for 48 h. The sequences of the used siRNA are: AllStars Negative Control siRNA (Qiagen, Germantown, MD, USA, 1027280) siSTAT3.3 (Qiagen, SI00048377) siSTAT3.4 (Qiagen, SI00048384).

Primary melanocytes were transduced with two *STAT3* shRNAs two times for each 12 h followed by two times transduction with *NRAS* mutants. The sequence of shRNAs used are: shRNA non targeting Control—(Dharmacon, Horizon Discovery, Lafayette, CO, USA, RHS4346), shSTAT3.1—TACCTAAGGCCATGAACTT (Dharmacon, V2LHS_88502), shSTAT3.2—ATAGTTGAAATCAAAGTCA (Dharmacon, V3LHS_376016).

### 4.4. Senescence Quantification

Transduced primary melanocytes were stained using Senescence β-Galactosidase Staining Kit (Cell Signaling Technology, Danvers, MA, USA, 9860) according to the manufacturer’s instructions. Number of β-Galactosidase positive cells was quantified and related to total number of cells by manual counting using brightfield micrographs after 0, 2, 6 and 10 days. The number of vacuolized cells was also quantified independently using brightfield micrographs. 

For detection of senescence-associated heterochromatin foci (SAHF) cells were fixed with 4% paraformaldehyde for 8 min. After three washes with PBS fixed cells were permeabilized with 0.1% Triton-X100 for 10 min, washed and stained with DAPI (Roche Diagnostics, Mannheim, Germany, 10 236 276 001, 1:2000, in TBST) for 5 min. Before imaging cells were washed 3 times with PBS. SAHF were quantified with ImageJ integrated cell counter and manual counting.

### 4.5. qPCR

Quantitative PCR was performed using SYBR™ Green PCR Master Mix (Applied Biosystems™, Foster City, CA, USA, 4309155) and 7500 Fast Real-Time PCR System (Applied Biosystems™). RNA from samples was isolated with RNeasy Mini Kit (Qiagen, 74106) and cDNA was synthesized. CT values were normalized to 18S as a housekeeping gene and relative expression of genes was quantified by calculating ΔΔCT. Primers were designed and validated with melting curve analysis. Following primer sequences were used: 18S FWD: GAGGATGAGGTGGAACGTGT, 18S REV: TCTTCAGTCGCTCCAGGTCT, IL1B FWD: TGTGAAATGCCACCTTTTGA, IL1B: GGTCAAAGGTTTGGAAGCAG, IL24 FWD: GACTTTAGCCAGCAGACCCTT, IL24 REV: GGTTGCAGTTGTGACACGAT, MMP2 FWD: TACAGGATCATTGGCTACACACC, MMP2 REV: GGTCACATCGCTCCAGACT, cMYC FWD: CTCCTCCTCGTCGCAGTAGA, cMYC REV: GCTGCTTAGACGCTGGATTT.

### 4.6. Western Blot

Cells were lysed and scraped with 10% Triton-10X, cOmplete™ Mini Protease Inhibitor Cocktail (Roche, Applied Science, Mannheim, Germany, 11836153001) and PhosSTOP™ (Roche, PHOSS-RO). Protein concentration was measured using Pierce™ BCA Protein Assay Kit (ThermoScientific™, Rockford, IL, USA, 23225) and 30 µg protein was loaded and separated on an SDS-PAGE Gel, then wet blotted on a 0.45 µm PVDF membrane (Merck, IEVH00010). The membranes were blocked and incubated in primary antibodies followed by an HRP-linked secondary antibody. The protein bands were visualized using Immobilon Forte Western HRP substrate (Merck, WBLUF) and Hyperfilm ECL (GE Healthcare, Chicago, IL, USA, 10607665), according to the manufacturer’s protocol. The primary antibodies used are: P-Stat3 (Y705, M9C6; CST 4113, 1:500 in 5% BSA), Stat3 (124 H6; CST 9139, 1:6000 in 5% BSA), β-Actin (13E5; CST 5125, 1:10 000 in 5% BSA), Phospho-p44/42 MAPK (T202/Y204, E10; CST 9106, 1:2000 in 5% Milk), p44/42 MAPK (Erk1/2,137F5; CST 4695, 1:4000 in 5% Milk), Phospho-Akt (Ser473, 193H12; CST 4058, 1:2000 in 5% Milk), Akt (40D4, CST 2920, 1:5000 in 5% Milk), mCherry (1C51; abcam, ab125096, 1:10000 in 5% Milk), α-Actinin (H-2; Santa Cruz, sc17829, 1:50000 in 5% Milk) and Axl (C44G1; CST 4566S, 1:1000 in 5% Milk).

### 4.7. Proliferation 

To measure proliferative ability, cells were plated at a density of 500 cells in a 96-well plate. After 0 and 9 days alamarBlue™ Cell Viability Reagent (Invitrogen™, ThermoFisher Scientific, DAL1100) was added and after 4 h of incubation at 37 °C, fluorescence was measured with excitation wavelength at 530–560 nm and emission wavelength at 590 nm with the Tecan Infinite 200 Pro plate reader. The fold increase of fluorescence intensity between day 0 and day 9 was calculated and plotted.

### 4.8. Colony Formation

200 cells were each plated in a 6 well-plate. Culture medium was changed every 2–3 days. After 11 days cells were fixed and stained with 0.05% Crystal Violet (1% formaldehyde (37%), 1% Methanol in PBS) for 20 min at room temperature. Cells were gently washed twice with tap water and air-dried. Area of the plate covered by stained colonies was quantified using the ImageJ Plugin ColonyArea [56].

### 4.9. Migration

To analyze the migratory potential, 35,000 cells were plated in each well of a 2-well-culture-insert (Ibidi, Planegg, Germany, 80209) with MEF-Medium. After cells attached to the plate 4 h later medium was incubated with FBS-free MEF-Medium overnight in the cell incubator. Inserts were removed the day after and cells were washed with PBS and 10% FBS MEF-Medium with 1 µg/mL Aphidicolin (Sigma-Aldrich^®^, A4487) was added. Cell migration was monitored after 8 h. TScratch Software was used for quantitative analysis of the closing gap.

### 4.10. Proteome Profiler Array

Protocol followed manual instructions from R&D Systems Europe, Ltd, Human Phospho-RTK Array Kit (#ARY001B) and Human Cytokine Array (#ARY005B). Briefly, cell lysates were diluted and incubated overnight with either array. The array was washed to remove unbound proteins followed by incubation with a cocktail of biotinylated detection antibodies and with streptavidin-HRP antibodies. Captured signal corresponded to the amount of bound phosphorylated protein.

### 4.11. Statistical Analyses

The statistical analyses of experiments were performed using the student’s *t*-test with a two-tailed distribution and homoscedasticity [57]. Data analysis was performed with GraphPad Prism (GraphPad Software Inc., San Diego, CA, USA). All experiments were performed at least in three biological replicates. Differences were considered as significant with a value of *p* < 0.05 (marked with *), *p* < 0.01 (marked with **), *p* < 0.001 (marked with ***) and *p* < 0.0001 (marked with ****).

## 5. Conclusions

In conclusion, we showed a clear contrast in the phenotypical behavior between NR*ASG12/13* and *NRASQ61*-mutated melanocytic cells. In primary melanocytes *NRASQ61* is able to override OIS through activation of the STAT3 pathway, whereas in immortalized melanocytes and melanoma cell lines, *NRASQ61* promotes a more tumorigenic behavior. Moreover, we identified in melanocytes a specific *NRASQ61*-driven senescence mechanism associated with the production of a set of cytokines and with the activation of kinases.

Furthermore, our study gives novel insights into molecular and cellular mechanisms, which are activated in response to *NRAS* oncogenic insult. For example, these results strongly suggest an implication of STAT3 in the context of the vast majority of *NRAS*-mutated melanoma patients who carry a codon 61 mutation and will help further validation of potential drug targets for this subgroup of patients.

## Supplementary Materials

The following are available online at https://www.mdpi.com/2072-6694/12/1/119/s1, Figure S1: (A) Cell proliferation assessed by alamar blue staining on MelSTV expressing *NRASWT*, *NRASG12V*, or *NRASQ61H* over 9 days. (B) Cell migration assay of MelSTV expressing *NRASWT*, *NRASG12V*, or *NRASQ61H*. (C) Colony formation assay of MelSTV expressing *NRASWT*, *NRASG12V*, or *NRASQ61H*. *n* = 3, *p* < 0.0001.
