# Supplementary material for: STAT3 Relays a Differential Response to Melanoma-Associated NRAS Mutations
Source: Cancers (Basel). 2020 Jan 2;12(1):119. doi: 10.3390/cancers12010119 (PMC7016650; doi:10.3390/cancers12010119)
Supplement: Supplementary file 1 [file cancers-12-00119-s001.pdf]

cancers

Supplementary Materials

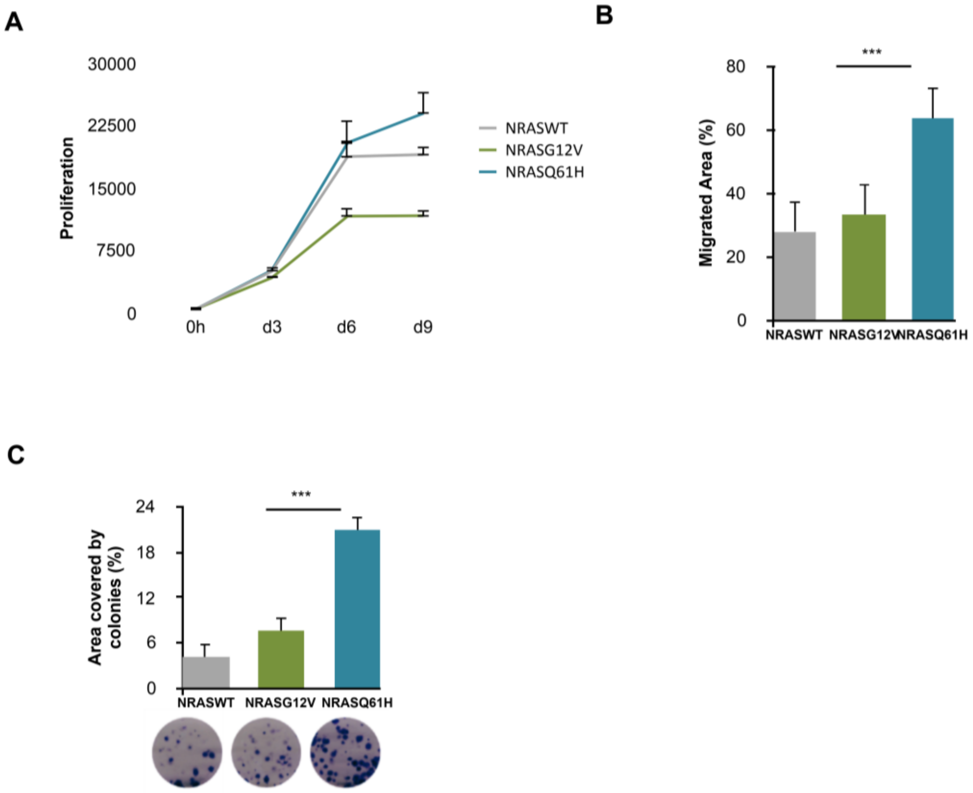

Figure S1. (**A**) Cell proliferation assessed by alamar blue staining on MelSTV expressing *NRASWT*, *NRASG12V*, or *NRASQ61H* over 9 days. (**B**) Cell migration assay of MelSTV expressing *NRASWT*, *NRASG12V*, or *NRASQ61H*. (**C**) Colony formation assay of MelSTV expressing *NRASWT*, *NRASG12V*, or *NRASQ61H*. $n = 3$, $p < 0.0001$.

Western Blot

Figure 2

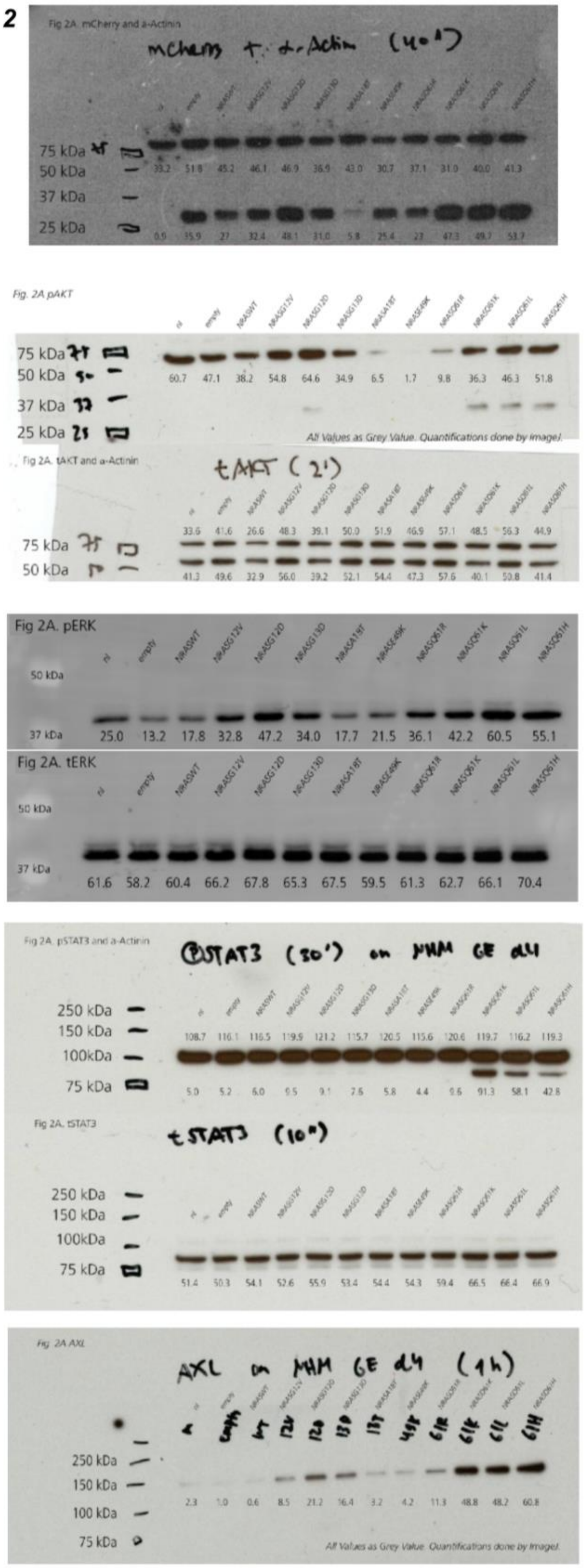

Figure 3

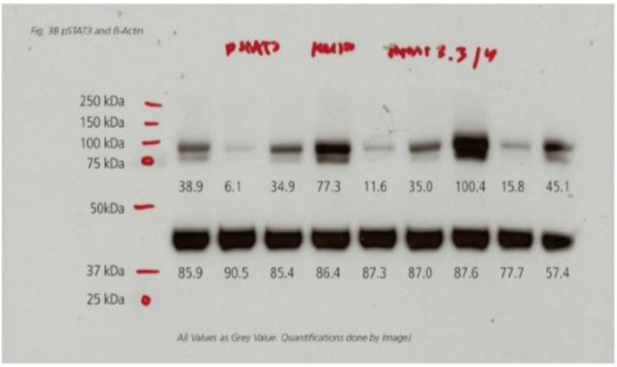
Fig. 3B pSTAT3 and B-Actin
250 kDa
150 kDa
100 kDa
75 kDa
38.9 6.1 34.9 77.3 11.6 35.0 100.4 15.8 45.1
50kDa
85.9 90.5 85.4 86.4 87.3 87.0 87.6 77.7 57.4
37 kDa
25 kDa
All Values as Grey Value. Quantifications done by ImageJ.

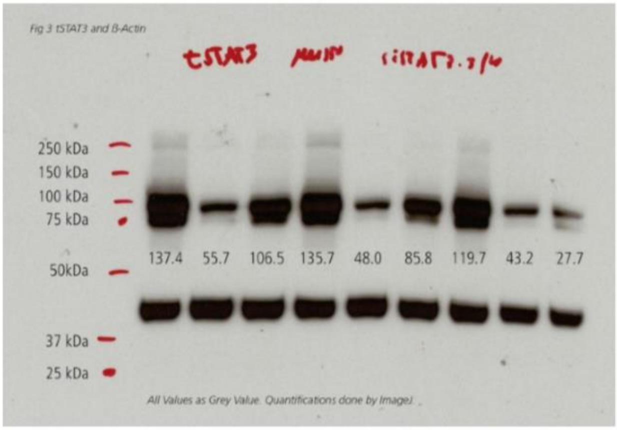
Fig 3 tSTAT3 and B-Actin
tSTAT3
250 kDa
150 kDa
100 kDa
75 kDa
137.4 55.7 106.5 135.7 48.0 85.8 119.7 43.2 27.7
50kDa
37 kDa
25 kDa
All Values as Grey Value. Quantifications done by ImageJ.

Figure 4

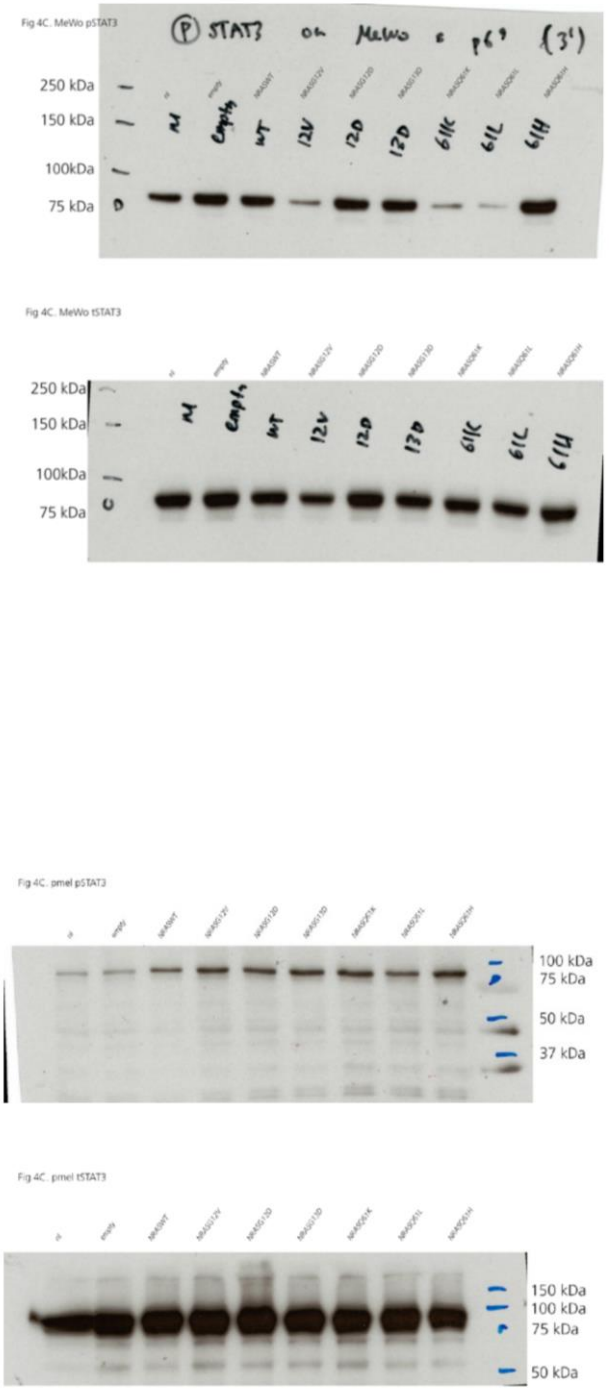

Fig 4C. MeWo pSTAT3
250 kDa
150 kDa
100kDa
75 kDa
Fig 4C. MeWo tSTAT3
250 kDa
150 kDa
100kDa
75 kDa
Fig 4C. pmel pSTAT3
100 kDa
75 kDa
50 kDa
37 kDa
Fig 4C. pmel tSTAT3
150 kDa
100 kDa
75 kDa
50 kDa
